# The potential value of disease-modifying therapy in patients with spinocerebellar ataxia type 1: an early health economic modeling study

**DOI:** 10.1007/s00415-023-11704-3

**Published:** 2023-04-19

**Authors:** Teije van Prooije, Sanne Ruigrok, Niels van den Berkmortel, Roderick P. P. W. M. Maas, Stan Wijn, Willeke M. C. van Roon-Mom, Bart van de Warrenburg, Janneke P. C. Grutters

**Affiliations:** 1 Department of Neurology, Donders Institute for Brain, Cognition and Behaviour, Radboud University Medical Center, Nijmegen, The Netherlands; 2 Department for Health Evidence, Radboud Institute for Health Sciences, Radboud University Medical Center, Nijmegen, The Netherlands; 3 Department of Operating Rooms, Radboud Institute for Health Sciences, Radboud University Medical Center, Nijmegen, The Netherlands; 4 Department of Human Genetics, Leiden University Medical Center, Leiden, The Netherlands

**Keywords:** Spinocerebellar ataxia type 1, Healthy economic modeling, Cost-effectiveness

## Abstract

**Objective:**

There currently is no disease-modifying therapy for spinocerebellar ataxia type 1 (SCA1). Genetic interventions, such as RNA-based therapies, are being developed but those currently available are very expensive. Early evaluation of costs and benefits is, therefore, crucial. By developing a health economic model, we aimed to provide first insights into the potential cost-effectiveness of RNA-based therapies for SCA1 in the Netherlands.

**Methods:**

We simulated disease progression of individuals with SCA1 using a patient-level state-transition model. Five hypothetical treatment strategies with different start and endpoints and level of effectiveness (5–50% reduction in disease progression) were evaluated. Consequences of each strategy were measured in terms of quality-adjusted life years (QALYs), survival, healthcare costs, and maximum costs to be cost effective.

**Results:**

Most QALYs (6.68) are gained when therapy starts during the pre-ataxic stage and continues during the entire disease course. Incremental costs are lowest (− €14,048) if therapy is stopped when the severe ataxia stage is reached. The maximum costs per year to be cost-effective are €19,630 in the “stop after moderate ataxia stage” strategy at 50% effectiveness.

**Discussion:**

Our model indicates that the maximum price for a hypothetical therapy to be cost-effective is considerably lower than currently available RNA-based therapies. Most value for money can be gained by slowing progression in the early and moderate stages of SCA1 and by stopping therapy upon entering the severe ataxia stage. To allow for such a strategy, it is crucial to identify individuals in early stages of disease, preferably just before symptom onset.

**Supplementary Information:**

The online version contains supplementary material available at 10.1007/s00415-023-11704-3.

## Introduction

Spinocerebellar ataxia type 1 (SCA1) is a rare, dominantly inherited neurodegenerative disease, within the heterogenous group of autosomal dominant cerebellar ataxias (ADCA). It is characterized by progressive cerebellar ataxia and is often accompanied by non-ataxia signs, such as spasticity and peripheral neuropathy [1]. SCA1 is caused by a CAG repeat expansion in the *ATXN1* gene [2], which results in a toxic gain of function of mutant ataxin-1 protein. The length of the expanded CAG repeat correlates with age of onset and rate of disease progression [2, 3].

Management of SCA1 is restricted to supportive care, including paramedical treatment and a limited number of drugs for symptom relief. Due to the disabling and progressive nature of SCA1, there is an urgent need of effective disease-modifying therapies. Previous studies have shown that downregulation of the mutant ataxin-1 protein via antisense oligonucleotide therapy (AON), specifically targeting ataxin-1 mRNA, can improve behavior and brain pathology in mouse models [4–8], suggesting a promising role for these drugs in patients with SCA1. Currently, preparations for clinical trials to test safety and efficacy of AON therapy in individuals with SCA1 are in an advanced stage.

The recent development and approval of AON-based drugs for infantile-onset spinal muscular atrophy showed that development of such an innovative therapy is a very costly process, resulting in high drug prices and pressure on the sustainability of health care systems [9]. AON therapy for SCA1 has been officially designated as an orphan drug in development due to the rarity of the disorder [10]. During the pricing of drugs such as AON therapy, not only the cost of research and development should be considered, but also its potential value for survival, quality of life, and society should be weighted.

Health economic modeling provides insight into the potential value of a healthcare intervention in its intended context, at an early stage of development [11]. It is rooted on the fact that in these early stages—although many uncertainties remain—development and research of healthcare innovation can still be (re)directed, without large financial or societal consequences. In later stages of development, redirecting innovation without consequences becomes increasingly difficult [12]. Health economic modeling synthesizes evidence on the current standard of care and compares this with a hypothetical care pathway that includes the innovation. This provides insight into the value for money and associated uncertainty, as well as informing on the efficient use of research and development to eventually implement and use the therapy to its full potential [13].

Here, we aimed to develop a health economic model that evaluates how a potentially disease-modifying treatment, such as AON therapy, can yield most added value for individuals with SCA1 and society in the perspective of the Dutch healthcare system, anticipating and informing the upcoming clinical trials. This model will inform developers, healthcare professionals, and all other parties involved, about the potential added value, future development, market access, and pricing of AON therapy as a treatment for SCA1.

## Methods

A patient-level state-transition model was constructed to simulate current practice, as well as the possibilities and consequences of a hypothetical future therapy in individuals with SCA1. The model simulates the consequences of disease progression of individual patients through different health states of SCA1, over a lifetime horizon.

The ISPOR/SMDM good research practices for modeling [14] were consulted for the development of the model and the CHEERS checklist was used for reporting [15].

### Population

Individuals with a SCA1 mutation were categorized according to their Scale for the Assessment and Rating of Ataxia (SARA) sum score, which ranges from 0 (indicating no ataxia) to 40 points (indicating very severe ataxia) [16]. The model simulates the disease course of 10,000 individuals with SCA1. We assumed that all individuals start in a pre-ataxic stage, with a SARA score of 0, and subsequently progress with an individual progression rate that was set to 1.83 SARA points increase every year (95% CI 1.45–2.21), as determined in a recent meta-analysis [17].

### Model structure

**Fig. 1:**
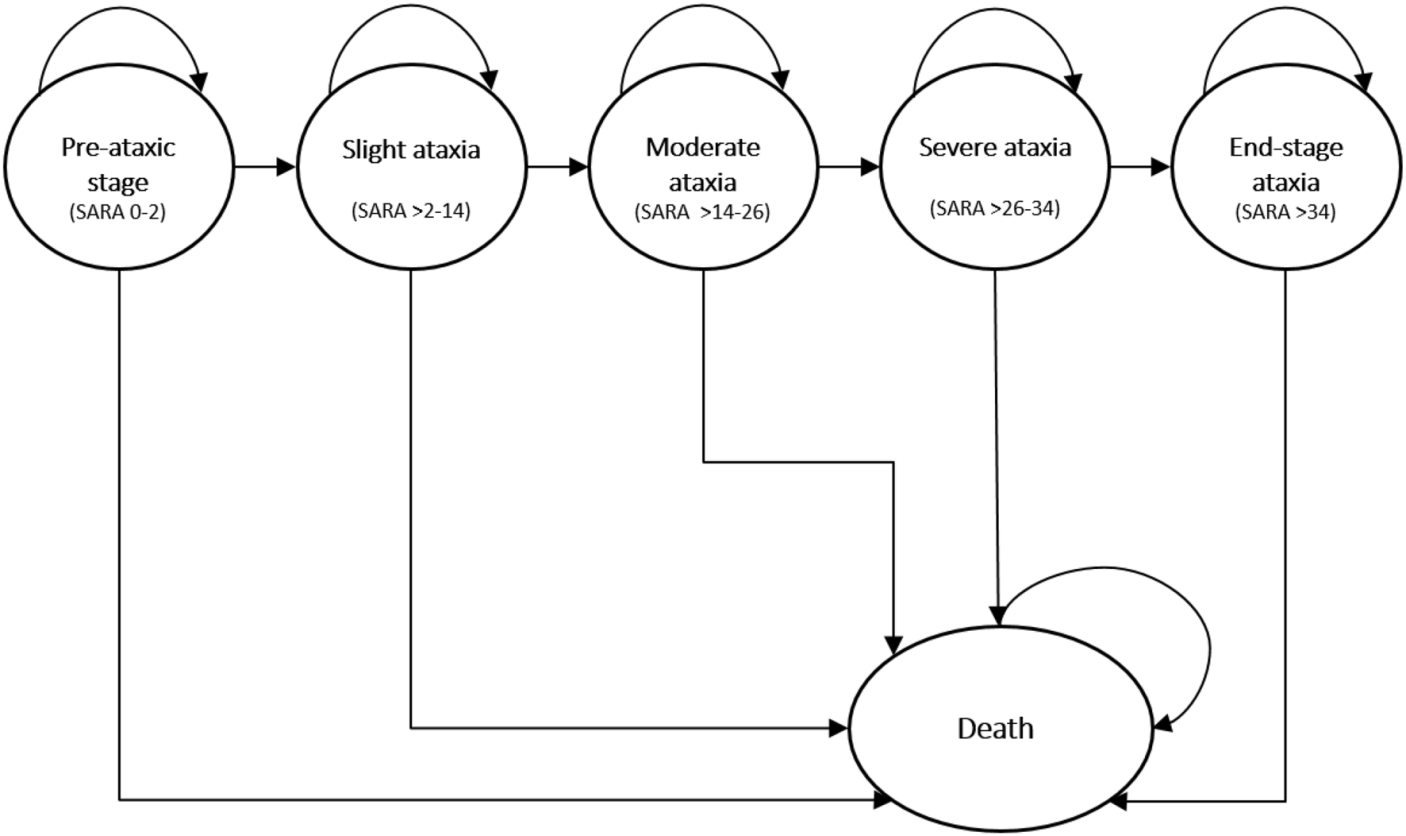
Conceptual overview of the state-transition model: depending on SARA score, individuals in the simulation model will either remain in their current health state or progress to the next health state. For all health states, there is also a probability to transition to the death state

In the model, individuals progress through different health states. These health states are based on SARA score: pre-ataxic (SARA 0–2), slight ataxia (SARA > 2–14), moderate ataxia (SARA > 14–26), severe ataxia (SARA > 26–34), and end-stage ataxia (SARA > 34) (see Fig. 1) [18]. The final absorbing health state is death. Over a lifetime horizon, individuals transition between health states, depending on their SARA score progression. This score changes with each yearly cycle. An individual can only be in one health state at the time, and there is the possibility of remaining in the same health state for multiple years. Due to the progressive nature of SCA1, we assumed that it is impossible to regress to a previous state.

### Transition probabilities

The individual progression rate was sampled from the mean SCA1 progression rate of 1.83 SARA points per year (95% CI 1.45–2.21) [17]. Mortality probability for individuals with SARA scores below 14 was based on Dutch population norms for age 35–44 [19]. For SARA scores of 14 and higher, 5-year survival rates were retrieved from the nomogram by Diallo et al. (see Supplementary Appendix 1a) [20]. These were converted to a 1-year mortality probability [21]. Age was not included in our calculation of the mortality probability, because the nomogram suggests that age has negligible impact on the survival probability of individuals with SCA1. We assumed that individuals with a SARA score of 40 died within 1 year.

### Therapeutic strategies

In addition to the usual care strategy (i.e., no targeted treatment and only symptom relief through medication and physical therapy), five alternative hypothetical strategies were modeled, in which individuals were simulated to receive a disease-modifying therapy with different start- and endpoints. To explore the effects of stopping therapy early after progression to more advanced disease stages, we included two strategies with earlier treatment endpoints.

The hypothetical treatment strategies were:

1. “Full treatment”—Therapy starts in the pre-ataxic stage, and continues for the rest of an individual’s life.
2. “Start at slight”—Therapy starts in the slight ataxia stage (SARA > 2), and continues for the rest of an individual’s life.
3. “Start at moderate”—Therapy starts in the moderate ataxia stage (SARA > 14), and continues for the rest of an individual’s life.
4. “Stop after moderate”—Therapy starts in the pre-ataxic stage, and continues until the end of the moderate ataxia stage (SARA = 26).
5. “Stop after severe”—Therapy starts in the pre-ataxic stage, and continues until the end of the severe ataxia stage (SARA = 34).

The effectiveness of AON therapy was modeled as a reduction in SARA progression rate. Previous animal model studies showed that AON therapy might be around 50% effective in terms of reducing levels of the mutant protein in relevant brain areas [22]. It is unclear how this reduction in mutant protein will translate to clinical outcomes. To model this uncertain effectiveness, we assumed that (1) AON treatment would not result in consistent improvement of SARA scores and that (2) 50% reduction on a molecular level maximally translates to an expected disease-modifying treatment effect of 50% reduction in SARA progression. The mean progression rate of individuals with SCA1 was therefore reduced with 5–50% (in incremental steps of 5%) in all five treatment strategies. We also assumed that effectiveness dropped to 0% directly after stopping therapy. All strategies were simulated ten times, once with every reduction percentage, to evaluate how different treatment effectiveness levels influence the potential value. It was assumed that all individuals are eligible for therapy and that the effectiveness of therapy is the same for all individuals and constant during treatment.

### Effects

The effects of each hypothetical treatment strategy were calculated in terms of survival and quality-adjusted life years (QALYs). QALYs combine both quality and duration of life. Quality of life is measured in terms of health state utility, which ranges from 0 (dead) to 1 (perfect health). One QALY can be translated to 1 year in perfect health.

To model QALYs of individuals with SCA1, utility scores for every health state were retrieved from a dataset of the EUROSCA study. This dataset contains SARA scores and corresponding EQ-5D-3L outcomes of 117 SCA1 patients, collected at 479 visits [23, 24]. Utility values of each visit were determined using the R package eq5d [25] using the index value set for the Netherlands [26]. The mean and 95% confidence interval of the utility value were calculated for patients with slight ataxia (*n* = 186), moderate ataxia (*n* = 193), severe ataxia (*n* = 77), and end-stage ataxia (*n* = 23) (see Table 1).

**Table 1:** Utility values per health state in SCA1

	Deterministic utility	95% CI	Source
Pre-ataxic	0.94	–	[26]
Slight ataxia	0.74	[0.72; 0.77]	[24]^a^
Moderate ataxia	0.65	[0.62; 0.67]	[24]^a^
Severe ataxia	0.38	[0.32; 0.45]	[24]^a^
End-stage ataxia	0.16	[0.06; 0.26]	[24]^a^

Values are based on EQ-5D data from the EuroSCA dataset. The utility value for the pre-ataxic stage is based on the Dutch EQ-5D-3L population norm at age 35–44

^a^Utility scores were calculated from the raw data of this study

The EUROSCA dataset did not include patients in the pre-ataxic stage. Considering the low impact of the disease in terms of symptoms in the earliest disease state, it was assumed that the utility value for this stage was equal to the Dutch EQ-5D-3L population norm at age 35–44, which includes the mean age of onset of 37 for SCA1 [26]. To determine QALYs, the utility values were multiplied by the number of years spent in that specific health state. To take time preferences into account, future QALYs were discounted at 1.5% annually according to Dutch guidelines [27].

### Costs

Costs included in the model consisted of healthcare expenditures per health state. An estimation of resource use (e.g., GP visits, outpatient clinic visits, physical therapy, and medication) was—in the absence of such data for SCA1—based on data of a small SCA3 patient cohort [28, 29] in consultation with two neurologists. Unit costs for these expenditures were derived from the Dutch guideline for costing research [27].

Healthcare expenditures relating to only a subset of patients (e.g., 20% receives home care in the moderate ataxia phase) were included as a weighted mean into the total costs of a health state. Where needed, costs were converted to the year 2020, using the consumer price index from Statistics Netherlands [30]. Costs per health state are presented in Table 2. To take time preferences into account, future costs were discounted at a 4% rate annually [27].

**Table 2:** Mean costs per health state per year

Resource	Unit cost	Resource use	% of patients	Mean cost per year
Pre-ataxic stage				
General practitioner	€37.00	1.5	100%	€55.50
Neurologist	€110.00	0.5	100%	€55.00
Total				€110.50
Slight ataxia				
Medication	€2.41	365	33%	€290.28
General practitioner	€37.00	1.5	100%	€55.50
Occupational physician	€28.00	1.5	100%	€42.00
Physical therapy	€37.00	39	100%	€1443.00
Neurologist	€110.00	1	100%	€110.00
Rehabilitation physician	€101.00	1	100%	€101.00
Total				€2041.78
Moderate ataxia				
Medication	€2.41	365	33%	€290.28
General practitioner	€37.00	1.5	100%	€55.50
Occupational physician	€28.00	1.5	100%	€42.00
Physical therapy	€37.00	52	100%	€1924.00
Speech therapy	€33.00	9	100%	€297.00
Occupational therapy	€37.00	1.5	50%	€27.75
Neurologist	€110.00	1	100%	€110.00
Rehabilitation physician	€101.00	1	100%	€101.00
Partner/family/friends	€15.50	390	33%	€1994.85
Home care	€77.50	312.5	20%	€4843.75
Total				€9686.13
Severe ataxia				
Medication	€2.41	365	33%	€290.28
General practitioner	€37.00	1.5	100%	€55.50
Physical therapy	€37.00	78	100%	€2886.00
Speech therapy	€33.00	9	100%	€297.00
Occupational therapy	€37.00	1.5	50%	€27.75
Neurologist	€110.00	1	100%	€110.00
Rehabilitation physician	€101.00	1	100%	€101.00
Partner/family/friends	€15.50	806	75%	€9369.75
Home care	€77.50	312.5	80%	€19,375.00
Nursing home care	€186.00	365	10%	€6789.00
Total				€39,301.28
End-stage ataxia				
Medication	€2.41	365	33%	€290.28
General practitioner	€37.00	1.5	100%	€55.50
Partner/family/friends	€15.50	1558	10%	€2414.90
Home care	€77.50	312.5	10%	€2421.88
Nursing home care	€186.00	365	90%	€61,101.00
Total				€66,283.56

The costs represent the estimated mean per patient in a particular health state per year. For all resources, a mean unit cost was calculated as well as a yearly average use of this resource. Medication unit is a dose, a unit of care by partner/family/friends is per hour, home care and nursing home units are per day, and all other resource units are per visit.

### Analysis

To explore the potential added value of a new therapy, we compared the five different treatment strategies with the usual care strategy by calculating the expected mean costs, mean years of survival after disease onset, years in each health state, and QALYs per patient. In addition, we calculated incremental differences between the usual care strategy and each of the hypothetical treatment strategies over different levels of effectiveness. In addition to this, a 95% confidence interval (CI) was calculated for all results, based on the simulation of 10,000 individuals.

Subsequently, we calculated the incremental net monetary benefit (iNMB) of each strategy compared to the usual care strategy at different effectiveness levels (5–50%). The iNMB was calculated by multiplying the incremental QALYs by the cost-effectiveness threshold, after which incremental costs are subtracted. For SCA1 in the Netherlands, the threshold was calculated to be €80,000 per QALY [31, 32]. Since the costs of the hypothetical therapy are not included in the model, the iNMB represents the maximum costs of the hypothetical therapy to be considered cost-effective. Higher iNMB values translate to a higher potential price of a therapy. To explore the effects of starting late in the disease course versus stopping early in the disease course, we calculated the maximum costs per year of treatment (yearly iNMB), by dividing the total iNMB by the mean years of treatment per strategy.

To explore the impact of uncertain parameters on the iNMB, deterministic one-way sensitivity analyses were performed. These sensitivity analyses provide insight into the extent to which a parameter influences the results of the model [33]. The impact of changing the following parameters was analyzed: healthcare expenses for the different health states (variation of 20% around the mean), progression rate of the usual care strategy, and utility values for the different health states (both varied along a 95% confidence interval). The resulting iNMB (at 50% effectiveness) for each changed parameter for the “Full treatment” strategy compared with the usual care strategy is presented in a tornado diagram. In addition, a scenario analysis with discount rates set to 0% and 5% was performed.

The model and plots were built in R (v4.1.0, The R Foundation for Statistical Computing, Vienna, Austria), using packages eq5d (version 0.10.1) and dplyr (version 1.0.7). In addition, the script was converted into a shiny dashboard using packages shiny (version 1.6.0) and shinydashboard (version 0.7.1) [34, 35]. The shiny dashboard is a user-friendly interactive tool in which other researchers and developers can change parameters and run the model, according to their jurisdiction and/or data on SCA1. The online shiny dashboard is available here: https://sruigrok.shinyapps.io/SCA1-CEA/

### Validation

The model was validated in accordance with the AdViShe checklist [36]. Face validity of the conceptual model and input parameters were checked by members of the team to see if it was suitable for SCA1 and the objective of this study. The computerized model was checked through extreme value testing and validated by an R-modeling expert. Operational validation in terms of outcomes was checked by face validity with the other members in the team.

### Standard protocol approvals, registrations, and patient consents

With regard to the EuroSCA data, the ethics committees of the participating centers (see Supplementary Appendix 2 for list of co-investigators) approved the study. The SCA3 medical consumption data were obtained through a study approved by the ethics committee of Radboud university medical center (NL65454.091.18). Informed consent from all these participants was obtained. All other information was derived from publicly available sources.

## Results

### Progression and survival

**Fig. 2:**
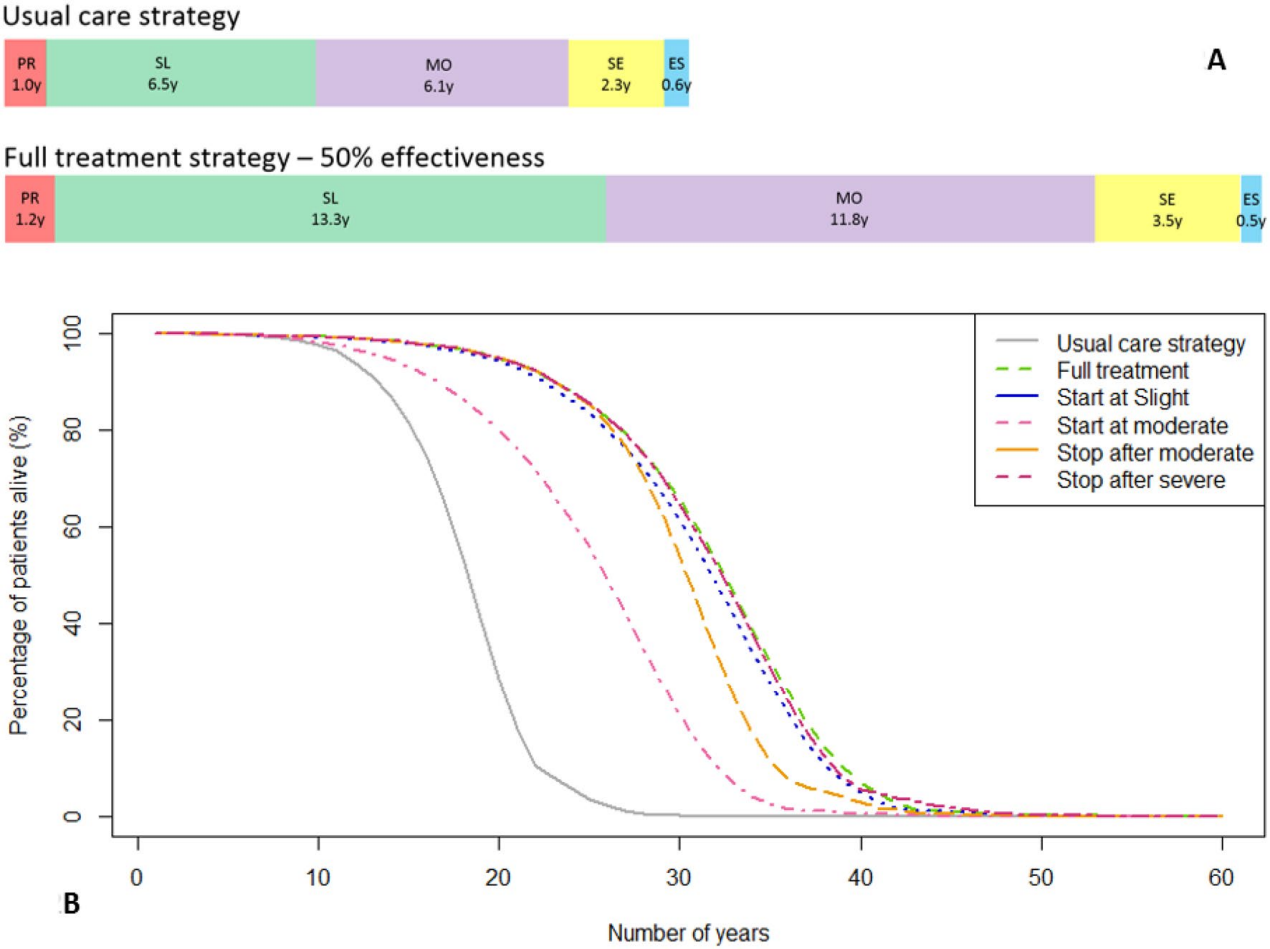
Survival in years of individuals with SCA1: **a** mean duration in years in the five different health states, comparing the usual care strategy and “Full treatment” strategy (at 50% effectiveness). *PR* pre-ataxic stage, *SL* slight ataxia, *MO* moderate ataxia, *SE* severe ataxia, *ES* end-stage ataxia. **b** Survival rate in years for individuals with SCA1 per treatment strategy at 50% effectiveness

The expected mean survival of individuals in the usual care strategy was 16.59 years (95% CI 16.51–16.66 years) after disease onset. Survival and the average time individuals spent in the health states slight, moderate, and severe ataxia were considerably longer if treated with a disease-modifying therapy with 50% effectiveness (see Fig. 2a). This delay in disease progression increased survival, but consequently also the probability of these individuals dying in an earlier disease stage. Therefore, the percentage of individuals reaching end-stage ataxia is lower for the “Full treatment” strategy compared to the usual care strategy, and the mean time spent in this health state for all individuals is shorter.

All hypothetical treatment strategies result in higher survival rates compared to the usual care strategy (see Fig. 2b). The largest gain in survival was 13.59 years (95% CI 13.46–13.72), with the “Full treatment” strategy. There was only a slight difference in survival for the different hypothetical treatment strategies, except for the “Start at moderate” strategy. At an effectiveness level of 50%, the mean survival for the hypothetical treatment strategies ranged from 23.57 years (95% CI 23.45–23.69) for the “Start at moderate” strategy to 30.18 years (95% CI 30.05–30.31) for the “Full treatment” strategy (see Supplementary Appendix 1b).

### Value for money

The usual care strategy yielded, on average, 10.44 QALYs (see Table 3) per individual. The expected gain in QALYs in the hypothetical treatment strategies ranged from 0.17 QALYs (95% CI 0.14–0.20; “Start at moderate” strategy, 5% effectiveness) to 6.68 QALYs (95% CI 6.63–6.72; “Full treatment” strategy, 50% effectiveness) per individual.

**Table 3:** Incremental costs, QALYs, and the iNMB of five different treatment strategies compared to the usual care strategy at 25% and 50% effectiveness

	25% effectiveness			50% effectiveness		
	Incremental costs	Incremental QALYs	iNMB	Incremental costs	Incremental QALYs	iNMB
Usual care	€118,821	10.44	**–**	€118,821	10.44	–
Full treatment	€5079	2.44	€190,165	€324	6.68	€534,274
Start at slight	€6217	2.31	€178,648	€3,385	6.27	€498,372
Start at moderate	€11,872	1.12	€77,734	€27,241	3.11	€221,557
Stop after moderate	€− 3604	2.21	€180,754	€− 14,048	6.15	€505,732
Stop after severe	€3686	2.42	€189,598	€− 755	6.65	€533,115

At 50% effectiveness, the incremental costs range from €14,048 saved (“Stop after moderate”) to extra costs of €27,241 per individual (“Start at moderate”). At 25% effectiveness, both incremental costs saved (€3604, at “stop after moderate”) and extra costs per individual (€11,872, “Start at moderate”) are lower compared to 50% effectiveness (see Table 3). Interestingly, for the “Stop after moderate” strategy, costs were lower compared to the usual care strategy for every effectiveness level. The only other treatment strategy that saved money (€755) compared to the usual care strategy was “Stop after severe” at 50% effectiveness (see Supplementary Appendix 1b).

The numbers for the usual care strategy are absolute costs and QALYs. A complete list with costs, QALY and survival in years at the different effectiveness levels for the hypothetical strategies is found in Supplementary Appendix 1b.

**Fig. 3:**
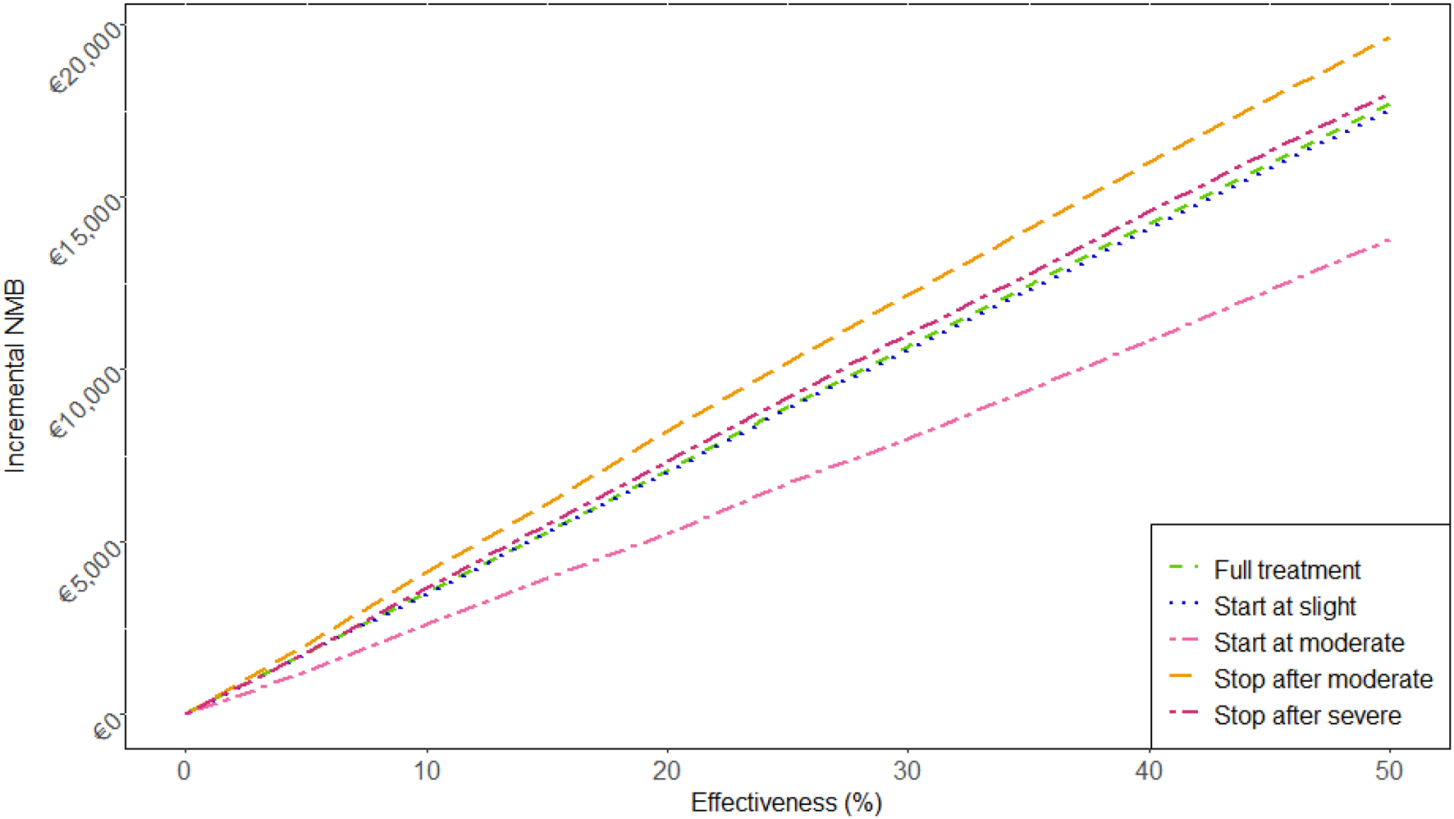
Incremental net monetary benefit (iNMB) per year of treatment: iNMB is modeled for for the different therapeutic strategies at various effectiveness levels ranging from 5 to 50%

The resulting total iNMB for the “Full treatment” strategy was highest (€534,274) at 50% effectiveness. This implies that if treatment is 50% effective, it is cost-effective if the total costs of therapy do not exceed €534,274. At 25% effectiveness iNMB was €190,165. The “Start at moderate” strategy had the lowest total iNMB for both 25% effectiveness (€77,734) and 50% effectiveness (€221,557) (see Table 3). If the iNMB is calculated per year of active treatment, the “Stop after moderate” strategy yields the highest iNMB. The corresponding maximum price per treatment year is €19,630 at 50% effectiveness in this strategy. Hence, the “Stop after moderate” strategy seems to provide most value for money compared to the other strategies (see Fig. 3).

### Deterministic one-way sensitivity analysis

**Fig. 4:**
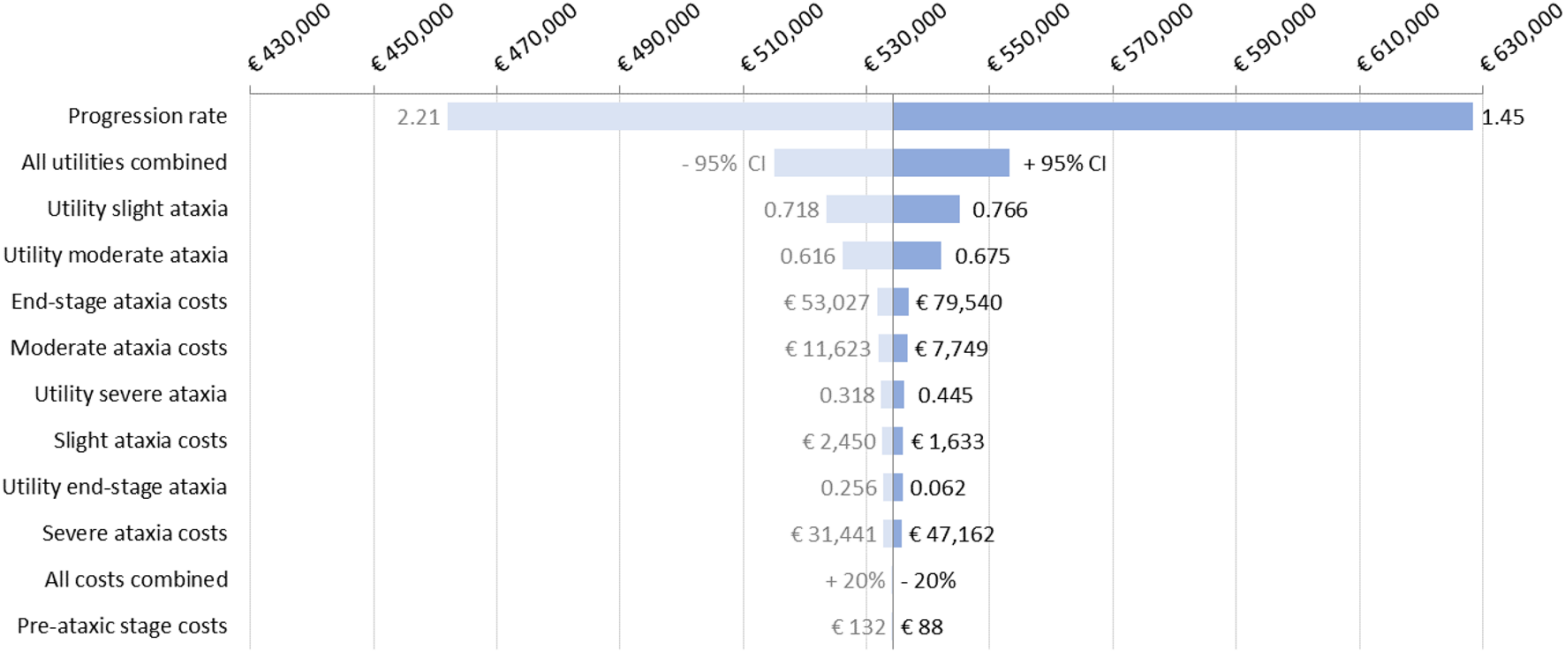
One-way sensitivity analysis: the tornado diagram provides a visualization of the impact of different parameters on the iNMB, with the most impactful parameters at the top. The analysis is performed for the “Full treatment” strategy at 50% effectiveness. The variation in parameters is presented at the side of the bars. Costs were varied 20% around the mean and utility and progression were varied as 95% CI around the mean

The results of the one-way sensitivity analyses are presented in Fig. 4. The effect of changing the different parameters on the total iNMB is shown for the “Full treatment” strategy at 50% effectiveness, which was found to be €534,274 in the base case. Changes in the modelled progression rate have the largest effect on the iNMB; from €628,499 at a 1.45 SARA point increase per year to €461,959 at 2.21 SARA point increase per year.

In an additional scenario analysis, the discount rate was varied for QALYs and costs from 0 to 5%. Variation of discounting of QALYs resulted in an iNMB change from €740,723 (0% discount) to €262,634 (5% discount). Discounting of costs has the opposite effect on the iNMB. A 0% discount rate results in an iNMB of €431,643 and the iNMB increases to €542,524 when the costs are discounted by 5%. No discounting of both costs and QALYs results in a total iNMB of €638,092 while 5% discounting of both results in an iNMB of €270,884.

## Discussion

We have constructed a health economic model to simulate the potential consequences of a disease-modifying therapy at different effectiveness levels and different treatment strategies for individuals with SCA1.

In the most optimistic scenario, where the biological effect of reducing mutant protein as established in animal models, translates to a therapeutic effect of 50% reduction in disease progression, our model suggests that most expected QALYs (6.68) are gained if treatment is started during the pre-ataxic stage (SARA ≤ 2 points) and continues for the rest of an individual’s life. Slower progression results in increased survival and a delay or prevention of transition into the more severe disease stages. Consequently, less individuals will reach end-stage ataxia in the “Full treatment” strategy compared to the usual care strategy. This is beneficial, since end-stage ataxia is associated with a high burden of disease, inevitable dependence on care and a substantially lower health-related quality of life (HRQOL) compared to earlier disease stages.

Additionally, the model suggests that, independent of effectiveness level, starting treatment as early as possible and stopping treatment after moderate ataxia provides most value for money in terms of total iNMB. The maximum price per treatment year at 50% effectiveness would be €19,630 in this scenario. Identical treatment strategies, but lower effectiveness levels would translate to lower total iNMB. Starting treatment at moderate ataxia and continuing for the rest on an individual’s life seems to provide least value for money at all effectiveness levels. The observed difference in iNMB between these two strategies can be explained by a relatively high HRQOL of individuals in the pre-ataxic and slight ataxia stages compared to individuals in later stages. Therefore, slowing progression in early ataxic stages has a greater effect on QALYs gained. If therapy is started in a more severe disease stage, this potential benefit is missed. Additionally, the results suggest that stopping the therapy before cost of illness becomes very high (from severe ataxia onwards) has similar socio-economic advantages as starting the therapy early.

### Strengths and limitations

Study strengths include the triangulation of data extraction, in which different studies and expert opinion were synthesized to construct an extensive model informing on a therapeutic strategy that provides most value for money. Another strength is the constructed shiny version of the simulation model. This shiny dashboard yields the possibility to easily incorporate and see the impact of further evidence on new therapies for SCA1 and other SCA subtypes, by serving as a template health economic model with adaptable parameters.

The study has several limitations. First, a fixed mean annual progression rate of 1.83 SARA points per year (95% CI 1.45–2.21) was used for the usual care strategy throughout the model, neglecting the possibility that progression might not be linear throughout the disease course and neglecting the well-known clinical heterogeneity of SCA1 and other factors potentially influencing individual progression rates, such as CAG repeat expansion size and individual fluctuations in SARA scores. Furthermore, the defined health states in the model, were based on SARA cut-offs derived from the literature. SARA is currently the best validated measure of disease severity, but the cut-offs used in the model might not reflect actual relevant patient-oriented disease milestones or transition from ambulant to non-ambulatory status. Including individual disease modifiers (such as CAG repeat length) and including validated patient-oriented outcomes as additional parameters might improve the validity of the model.

Second, effectiveness of AON therapy is uncertain, but was now modeled the same for all individuals, and was constant over time. However, it is unknown if AON therapy and other genetic interventions are effective in all disease stages and if clinical efficacy is similar for all patients.

Third, a Dutch perspective was used to support the model and its parameters, such as costs and discounting, limiting its representativeness of SCA1 globally. By performing sensitivity analyses, and conversion of the model into a shiny version, which enables researchers to change costs and discount rates according to their jurisdiction, this limitation was countered.

A final limitation of this model concerns the scarce literature and data available on utility and costs of individuals with SCA1. Our model is based on data from small cohort studies. Consequently, the variables in the model do contain uncertainties that affect the iNMB, visible in the one-way sensitivity analyses results. Societal costs, such as productivity loss, were not included in this model because no data were available. This might result in underestimation of the maximum costs of treatment, as we expect that nearly all individuals with a SARA score > 20 will be unable to work. Postponing this moment will likely further increase the iNMB and maximum price of treatment. A recent study capturing the situation in Ireland, estimated the costs of living with inherited ataxia around €60,000 per person, per year, much higher than the costs that were estimated for slight, mild, moderate and severe ataxia patients in this model. Indirect costs, based on productivity losses, accounted for 52% of the total costs, indicating large potential impact on iNMB [37]. Further research is clearly needed to map costs and utilities associated with SCA1 and other SCAs in the Netherlands.

### Implications for clinical practice

Our model suggests that a strategy in which therapy is initiated in pre-ataxic mutation carriers, close to age of onset might be most cost-effective. For this scenario, pre-symptomatic genetic testing and accurate prediction models for age of ataxia onset, based on individual progression (bio)markers, are required as studies suggest that the pre-ataxic stage could potentially start up to 10 years prior to clinical onset of ataxia in SCA1 and other SCAs [38, 39].

In addition to an early start of the treatment, the model suggests that it is most cost-effective to stop treatment after the moderate ataxia stage. After this stage, the potential benefits of therapy seem to no longer outweigh the burden of the illness, in terms of utility and mortality probability. However, deciding to stop an effective therapy based on value for money, raises several ethical considerations, especially when individuals have perceived positive effects of the therapy.

Furthermore, our results suggest that cost-effectiveness (i.e., a cost-effectiveness threshold < €80,000 per QALY) can be achieved with a maximum treatment cost per year per patient of less than €20,000. This is considerably lower than a similar AON therapy, i.e. nusinersen for spinal muscular atrophy, which costs a €234 k per year [40]. However, the financial tipping point of cost-effectiveness is not the only factor that will determine reimbursement and implementation, as regulatory authorities will also take ethical aspects and societal impact into consideration when negotiating drug pricing with industry.

## Conclusion

This health economic model has provided important first insights into the value for money of disease-modifying, genetic therapies in SCA1, and, as such, may guide their development and research. The model suggests that the maximum price for these interventions to be cost-effective in SCA1 is considerably lower than prices of comparable treatments currently on the market for other rare inherited diseases. Most value for money can be gained by slowing progression in specifically the early and moderate stages of SCA1 (SARA < 26) and by stopping therapy upon reaching the severe ataxia stage. To allow for such a strategy, it will be crucial to identify individuals in pre-ataxic stages of disease and accurately predict time to symptom onset.

## Supplementary Information

Below is the link to the electronic supplementary material.

Supplementary file1 (DOCX 30 KB)
