# Correction to: The potential value of disease-modifying therapy in patients with spinocerebellar ataxia type 1: an early health economic modeling study

**DOI:** 10.1007/s00415-023-11751-w

**Published:** 2023-05-08

**Authors:** Teije van Prooije, Sanne Ruigrok, Niels van den Berkmortel, Roderick P. P. W. M. Maas, Stan R. W. Wijn, Willeke M. C. van Roon-Mom, Bart van de Warrenburg, Janneke P. C. Grutters

**Affiliations:** 1 Department of Neurology, Donders Institute for Brain, Cognition and Behaviour, Radboud University Medical Center, Nijmegen, The Netherlands; 2 Department for Health Evidence, Radboud Institute for Health Sciences, Radboud University Medical Center, Nijmegen, The Netherlands; 3 Department of Operating Rooms, Radboud Institute for Health Sciences, Radboud University Medical Center, Nijmegen, The Netherlands; 4 Department of Human Genetics, Leiden University Medical Center, Leiden, The Netherlands

**Correction to: Journal of Neurology** 10.1007/s00415-023-11704-3

The original version of this article unfortunately contained a mistake. The author’s name Stan R. W. Wijn was incorrectly written as Stan Wijn.

