# Supplementary material for: The potential value of disease-modifying therapy in patients with spinocerebellar ataxia type 1: an early health economic modeling study
Source: J Neurol. 2023 Apr 19;270(8):3788–98. doi: 10.1007/s00415-023-11704-3 (PMC10344992; doi:10.1007/s00415-023-11704-3)
Supplement: Supplementary file 1 — Supplementary file1 (DOCX 30 KB) [file 415_2023_11704_MOESM1_ESM.docx]

**Appendix 1a -** 1-year mortality probability (MP) for individuals with SCA1 based on the SARA score

|  | | | | | | | | | | |
| --- | --- | --- | --- | --- | --- | --- | --- | --- | --- | --- |
| SARA | **1** | **2** | **3** | **4** | **5** | **6** | **7** | **8** | **9** | **10** |
| *MP* | *0.000586* | *0.000586* | *0.000586* | *0.000586* | *0.000586* | *0.000586* | *0.000948* | *0.000948* | *0.000948* | *0.000948* |
| SARA | **11** | **12** | **13** | **14** | **15** | **16** | **17** | **18** | **19** | **20** |
| *MP* | *0.000948* | *0.000948* | *0.000948* | *0.002573* | *0.003626* | *0.004765* | *0.005909* | *0.007059* | *0.008214* | *0.009374* |
| SARA | **21** | **22** | **23** | **24** | **25** | **26** | **27** | **28** | **29** | **30** |
| *MP* | *0.011949* | *0.018147* | *0.024506* | *0.031036* | *0.037746* | *0.044649* | *0.051757* | *0.059085* | *0.066754* | *0.08539* |
| SARA | **31** | **32** | **33** | **34** | **35** | **36** | **37** | **38** | **39** | **40** |
| *MP* | *0.103221* | *0.13296* | *0.157291* | *0.189387* | *0.227557* | *0.27522* | *0.33505* | *0.40324* | *0.404324* | *0.404324* |

Values for SARA scores of 1-14 are based on the Dutch average mortality probability in age groups 35-39 and 40-44^20^. Values for SARA scores >14 are converted from the 5 year survival probability in Diallo et al ^21^.

**Appendix 1b -** Mean costs, QALY and survival in years for all five treatment strategies at effectiveness levels 5-50*%.*

|  | | | | | |
| --- | --- | --- | --- | --- | --- |
|  |  |  | **Strategy** |  |  |
|  | **Full treatment** | **Start at slight** | **Start at moderate** | **Stop after moderate** | **Stop after severe** |
| **Costs** | | | | | |
| 5% | € 120,153 [± € 1,368] | € 120,489 [± € 1,374] | € 120,822 [± € 1,394] | € 117,973 [± € 1,354] | € 119,873 [± € 1,368] |
| 10% | € 121,293 [± € 1,379] | € 121,832 [± € 1,382] | € 123,473 [± € 1,397] | € 117,923 [± € 1,332] | € 120,636 [± € 1,359] |
| 15% | € 121,712 [± € 1,378] | € 122,111 [± € 1,382] | € 125,794 [± € 1,424] | € 117,311 [± € 1,316] | € 121,484 [± € 1,342] |
| 20% | € 123,599 [± € 1,377] | € 123,913 [± € 1,374] | € 128,922 [± € 1,446] | € 116,056 [± € 1,274] | € 122,764 [± € 1,334] |
| 25% | € 123,901 [± € 1,353] | € 125,038 [± € 1,358] | € 130,693 [± € 1,452] | € 115,217 [± € 1,243] | € 122,507 [± € 1,310] |
| 30% | € 124,008 [± € 1,341] | € 125,607 [± € 1,353] | € 134,694 [± € 1,477] | € 113,398 [± € 1,201] | € 122,928 [± € 1,297] |
| 35% | € 123,609 [± € 1,324] | € 125,150 [± € 1,334] | € 137,830 [± € 1,484] | € 111,368 [± € 1,155] | € 121,890 [± € 1,261] |
| 40% | € 122,448 [± € 1,288] | € 124,649 [± € 1,293] | € 140,153 [± € 1,480] | € 109,640 [± € 1,098] | € 120,946 [± € 1,220] |
| 45% | € 120,748 [± € 1,241] | € 123,436 [± € 1,255] | € 142,395 [± € 1,485] | € 107,539 [± € 1,047] | € 119,829 [± € 1,182] |
| 50% | € 119,145 [± € 1,189] | € 122,207 [± € 1,201] | € 146,063 [± € 1,480] | € 104,774 [± € 981.0] | € 118,067 [± € 1,135] |
| **QALY** | | | | | |
| 5% | 10.85 [± 0.029] | 10.83 [± 0.029] | 10.61 [± 0.028] | 10.79 [± 0.028] | 10.83 [± 0.028] |
| 10% | 11.28 [± 0.030] | 11.23 [± 0.030] | 10.83 [± 0.029] | 11.20 [± 0.029] | 11.27 [± 0.030] |
| 15% | 11.74 [± 0.031] | 11.67 [± 0.031] | 11.05 [± 0.031] | 11.63 [± 0.031] | 11.75 [± 0.032] |
| 20% | 12.29 [± 0.033] | 12.18 [± 0.033] | 11.29 [± 0.032] | 12.12 [± 0.032] | 12.28 [± 0.033] |
| 25% | 12.88 [± 0.035] | 12.75 [± 0.035] | 11.56 [± 0.034] | 12.66 [± 0.033] | 12.86 [± 0.035] |
| 30% | 13.53 [± 0.037] | 13.35 [± 0.037] | 11.87 [± 0.035] | 13.25 [± 0.035] | 13.51 [± 0.037] |
| 35% | 14.25 [± 0.040] | 14.02 [± 0.039] | 12.21 [± 0.037] | 13.92 [± 0.037] | 14.22 [± 0.040] |
| 40% | 15.07 [± 0.042] | 14.81 [± 0.041] | 12.59 [± 0.040] | 14.69 [± 0.038] | 15.06 [± 0.042] |
| 45% | 16.02 [± 0.045] | 15.68 [± 0.044] | 13.02 [± 0.042] | 15.57 [± 0.041] | 16.00 [± 0.045] |
| 50% | 17.12 [± 0.048] | 16.71 [± 0.047] | 13.55 [± 0.045] | 16.59 [± 0.044] | 17.10 [± 0.048] |
| **Survival** | | | | | |
| 5% | 17.37 [± 0.076] | 17.34 [± 0.076] | 16.96 [± 0.075] | 17.21 [± 0.074] | 17.32 [± 0.075] |
| 10% | 18.19 [± 0.080] | 18.11 [± 0.079] | 17.43 [± 0.078] | 17.96 [± 0.077] | 18.16 [± 0.079] |
| 15% | 19.09 [± 0.083] | 18.96 [± 0.083] | 17.91 [± 0.081] | 18.74 [± 0.079] | 19.09 [± 0.083] |
| 20% | 20.18 [± 0.088] | 19.99 [± 0.087] | 18.45 [± 0.085] | 19.63 [± 0.082] | 20.16 [± 0.087] |
| 25% | 21.36 [± 0.093] | 21.15 [± 0.093] | 19.04 [± 0.090] | 20.64 [± 0.085] | 21.27 [± 0.092] |
| 30% | 22.67 [± 0.099] | 22.36 [± 0.098] | 19.75 [± 0.095] | 21.75 [± 0.089] | 22.59 [± 0.098] |
| 35% | 24.13 [± 0.105] | 23.73 [± 0.104] | 20.53 [± 0.100] | 23.00 [± 0.093] | 24.01 [± 0.104] |
| 40% | 25.81 [± 0.112] | 25.35 [± 0.111] | 21.37 [± 0.106] | 24.51 [± 0.097] | 25.73 [± 0.111] |
| 45% | 27.78 [± 0.120] | 27.19 [± 0.119] | 22.33 [± 0.113] | 26.25 [± 0.103] | 27.72 [± 0.120] |
| 50% | 30.18 [± 0.130] | 29.46 [± 0.129] | 23.57 [± 0.122] | 28.31 [± 0.110] | 30.08 [± 0.130] |
| For all results a 95% confidence interval is included. For the usual care strategy, costs are €118,821 [± €1,373]. QALYs are 10.44 [± 0.028] and survival in years is 16.59 [± 0.074]. | | | | | |

**Appendix 2 –** Coinvestigators (the EUROSCA study group)

| **Name** | **Location** | **Role** | **Contribution** |
| --- | --- | --- | --- |
| Heike Jacobi, MD | Department of Neurology, University Hospital of Bonn, Bonn, Germany | **Site investigator** | **Data collection** |
| Sophie Tezenas du Montcel, PhD | Sorbonne Universités, Université Pierre et Marie Curie (UPMC) Univ Paris 06, UMR S 1136, INSERM U 1136, Institut Pierre Louis d’Epidémiologie et de Santé Publique, F-75013, Paris, France and AP-HP, Biostatistics Unit, Groupe Hospitalier Pitié-Salpêtrière, F-75013, Paris, France | **Statistician** | **Data analysis** |
| Peter Bauer, MD | Institute of Medical Genetics and Applied Genomics, University of Tübingen, Tübingen, Germany | **Geneticist** | **Genetic testing of EuroSCA samples** |
| Paola Giunti, PhD | Department of Molecular Neuroscience, UCL, Institute of Neurology, London, United Kingdom | **Site investigator** | **Data collection** |
| Arron Cook, MBBS | Department of Molecular Neuroscience, UCL, Institute of Neurology, London, United Kingdom | **Site investigator** | **Data collection** |
| Robyn Labrum, MD | Neurogenetic Laboratory, National Hospital of Neurology and Neurosurgery, UCLH, London, United Kingdom | **Site investigator** | **Data collection** |
| Michael H. Parkinson, MBBS | Department of Molecular Neuroscience, UCL, Institute of Neurology, London, United Kingdom | **Site investigator** | **Data collection** |
| Alexandra Durr, PhD | INSERM, U 1127, F-75013, Paris, France, CNRS, UMR 7225, F-75013, Paris, France, Sorbonne Universités, UPMC Univ Paris 06, UMRS_1127, F-75013, Paris, France, Institut du Cerveau et de la Moelle épinière, ICM, F-75013, Paris, France and APHP, Hôpital de la Pitié-Salpêtrière, Département de Génétique, F-75013, Paris, France | **Site investigator** | **Data collection** |
| Alexis Brice, MD | INSERM, U 1127, F-75013, Paris, France, CNRS, UMR 7225, F-75013, Paris, France, Sorbonne Universités, UPMC Univ Paris 06, UMRS_1127, F-75013, Paris, France, Institut du Cerveau et de la Moelle épinière, ICM, F-75013, Paris, France and APHP, Hôpital de la Pitié-Salpêtrière, Département de Génétique, F-75013, Paris, France | **Co-coordinator** |  |
| Perrine Charles, MD | APHP, Hôpital de la Pitié-Salpêtrière, Département de Génétique, F-75013, Paris, France | **Site investigator** | **Data collection** |
| Cecilia Marelli, MD | Service de Neurologie – CMRR, CHRU Gui de Chauliac, 80, av. A. Fliche, 34295 - Montpellier CEDEX 05, France | **Site investigator** | **Data collection** |
| Caterina Mariotti, MD | SOSD Genetics of Neurodegenerative and Metabolic Diseases, Fondazione-IRCCS Istituto Neurologico Carlo Besta, Milan, Italy | **Site investigator** | **Data collection** |
| Lorenzo Nanetti, MD | SOSD Genetics of Neurodegenerative and Metabolic Diseases, Fondazione-IRCCS Istituto Neurologico Carlo Besta, Milan, Italy | **Site investigator** | **Data collection** |
| Marta Panzeri, MD | SOSD Genetics of Neurodegenerative and Metabolic Diseases, Fondazione-IRCCS Istituto Neurologico Carlo Besta, Milan, Italy | **Site investigator** | **Data collection** |
| Maria Rakowicz, PhD | Department of Clinical Neurophysiology, Institute of Psychiatry and Neurology, Warsaw, Poland | **Site investigator** | **Data collection** |
| Anna Sulek, PhD | Department of Genetics, Institute of Psychiatry and Neurology, Warsaw, Poland | **Site investigator** | **Data collection** |
| Anna Sobanska, MD | Department of Clinical Neurophysiology, Institute of Psychiatry and Neurology, Warsaw, Poland | **Site investigator** | **Data collection** |
| Tanja Schmitz-Hübsch, MD | Charité Universitätsmedizin Berlin, Klinik für Neurologie, Berlin, Germany | **Site investigator** | **Data collection** |
| Ludger Schöls, MD | Department of Neurodegeneration and Hertie-Institute for Clinical Brain Research, University of Tübingen and Deutsches Zentrum für Neurodegenerative Erkrankungen (DZNE), D-72076 Tübingen, Germany | **Site investigator** | **Data collection** |
| Holger Hengel, MD | Department of Neurodegeneration and Hertie-Institute for Clinical Brain Research, University of Tübingen and Deutsches Zentrum für Neurodegenerative Erkrankungen (DZNE), D-72076 Tübingen, Germany | **Site investigator** | **Data collection** |
| Laszlo Baliko, MD | Department of Neurology, Zala County Hospital, H-8900 Zalaegerszeg, Zrinyi M. Str. 1., Hungary | **Site investigator** | **Data collection** |
| Bela Melegh, PhD | Department of Medical Genetics, and Szentagothai Research Center, University of Pécs, Pécs, Hungary | **Site investigator** | **Data collection** |

| Alessandro Filla, MD | Department of Neuroscience, and Reproductive and Odontostomatological Sciences, Federico II University Naples, Italy | **Site investigator** | **Data collection** |
| --- | --- | --- | --- |
| Antonella Antenora, MD | Department of Neuroscience, and Reproductive and Odontostomatological Sciences, Federico II University Naples, Italy | **Site investigator** | **Data collection** |
| Jon Infante, MD | Service of Neurology, University Hospital Marqués de Valdecilla (IDIVAL), University of Cantabria (UC) and Centro de Investigación Biomédica en Red de Enfermedades Neurodegenerativas (CIBERNED), Santander, Spain | **Site investigator** | **Data collection** |
| José Berciano, MD | Service of Neurology, University Hospital “Marqués de Valdecilla (IDIVAL)", "Centro de Investigación Biomédica en Red de Enfermedades Neurodegenerativas (CIBERNED)", University of Cantabria (UC), Santander, Spain | **Site investigator** | **Data collection** |
| Dagmar Timmann, MD | Department of Neurology, University Clinic Essen, University of Duisburg-Essen | **Site investigator** | **Data collection** |
| Sandra Szymanski, MD | Department of Neurology, St. Josef Hospital, University Hospital of Bochum, Bochum, Germany | **Site investigator** | **Data collection** |
| Sylvia Boesch, MD | Department of Neurology, Medical University, Innsbruck, Innsbruck Austria | **Site investigator** | **Data collection** |
| Jun-Suk Kang, MD | Department of Neurology, University of Frankfurt, Frankfurt/M, Germany | **Site investigator** | **Data collection** |
| Massimo Pandolfo, MD | Université Libre de Bruxelles (ULB), Neurology Service - ULB Hôpital Erasme, ULB Laboratory of Experimental Neurology, Brussels, Belgium | **Site investigator** | **Data collection** |
| Jörg B. Schulz, MD | Department of Neurology, RWTH Aachen University, Pauwelsstraβe 30, 52074 Aachen, Germany and JARA - Translational Brain Medicine, Aachen-Jülich, INM 11, Germany | **Site investigator** | **Data collection** |
| Sonia Molho, Msc, | AP-HP, Biostatistics Unit, Groupe Hospitalier Pitié-Salpêtrière, F-75013, Paris, France | **Site investigator** | **Data collection** |

| Alhassane Diallo, MD | Sorbonne Universités, Université Pierre et Marie Curie (UPMC) Univ Paris 06, UMR S 1136, INSERM U 1136, Institut Pierre Louis d’Epidémiologie et de Santé Publique, F-75013, Paris, France | **Statistician** | **Data analysis** |
| --- | --- | --- | --- |
| Thomas Klockgether, MD | Department of Neurology, University Hospital of Bonn, Bonn, Germany and German Center for Neurodgenerative Diseases (DZNE), Bonn, Germany | **Coordinator** |  |
